# Assessing proprioception through time-variability properties of acceleration

**DOI:** 10.3389/fphys.2023.1112902

**Published:** 2023-01-20

**Authors:** Lluc Montull, Alex Borrallo, Maricarmen Almarcha, Natàlia Balagué

**Affiliations:** ^1^ Complex Systems in Sport Research Group, National Institute of Physical Education of Catalonia (INEFC), University of Lleida, Lleida, Spain; ^2^ Complex Systems in Sport Research Group, National Institute of Physical Education of Catalonia (INEFC), University of Barcelona, Barcelona, Spain

**Keywords:** detrended fluctuation analysis, kinematic variable, motor control, slackline, movement stability, balance, training

## Abstract

Proprioception is a crucial property for movement stability and balance, but its current assessment, based on clinical testing, lacks precision and adequacy in real contexts. This study proposes assessing proprioception and its sensitivity to training effects through acceleration time series recorded during two slackline experiments. In the first experiment, slackliners of different expertise (highly and poorly trained) had to walk on a slackline for 30 s. In the second, twelve beginners had to balance up on the slackline for at least 11 s before and after a training process. Acceleration time series were recorded in body components (legs and centre of mass) and the slackline. The acceleration fluctuations were analysed through Detrended Fluctuation Analysis. The obtained Hurst (H)-exponents were compared between both groups (first experiment) and before and after training (second experiment) using Whitney and Wilcoxon tests, respectively. The values of H-exponents were lower in the highly trained group (Z = −2.15, *p* = 0.03) (first experiment), and in the post-training conditions (Z = −2.35, *p* = 0.02) (second experiment). These results suggest better motor and proprioceptive control with training status. Hence, the time-variability structure of acceleration in real contexts, like slackline tasks, is proposed as an objective measure of proprioception and its training effects.

## 1 Introduction

Proprioception, the sensation of body position and movement, often described as the “sixth sense”, plays a crucial role in human motor control, performance, and injury prevention (Tuthill and Azim, 2018). Together with ocular, vestibular, or haptic sensitivity, it is essential in movement stability and balance tasks with dominant proprioceptive feedback (Ogard, 2011). Its objective assessment is difficult because the available methods, protocols, and analysis techniques, mainly applied to clinical contexts, lack precision and adequacy (Hillier et al., 2015).

Proprioception has been mostly assessed in the scope of a general clinical environment for “screening” the influence of impaired proprioception and guiding rehabilitation interventions (Hillier et al., 2015; Röijezon et al., 2015). Capturing the error or differences between the targeted and the performed movements, force sense tests, or threshold detection of passive motion stand as widely accepted examples of proprioception assessment (Lephart et al., 2002; Weerakkody et al., 2008; Han et al., 2016).

The proprioceptive tests used in clinical practice, based on conscious proprioception and decontextualized movements requiring reproduction or even verbalization of simple motions, are inappropriate to be applied in real settings (Ager et al., 2017; Muñoz-Jiménez et al., 2021). Unlike in clinical contexts, in daily activities, the sub-conscious proprioception is dominantly relevant (Hillier et al., 2015). For instance, in sports and exercise, proprioception contributes to the coordination and fine adjustments of body movements to maintain dynamic stability in safe conditions (Balagué et al., 2014; Vázquez et al., 2016; 2021). Such fine adjustments emerging from perception-action cycles, acting at very short timescales (i.e., actions instantaneously create new perceptions for future actions), cannot be consciously captured (Shaw and Kinsella-Shaw, 2007; Montull et al., 2020).

In addition, proprioceptive standard measurements, often contrasted with theoretical group-pooled data to categorize a prototypic “healthy” state, neglect the uniqueness and richness of individual adaptive responses under changing real constraints (Hillier et al., 2015). Then, the use of activities like slacklining, skiing, or acrobatics, which require balance and stability, can be useful in assessing individual proprioceptive properties in real settings. The registration of time series of kinematic variables extracted from body components and interacting instruments (e.g., slackline) during these activities may be seen as promising measures to capture such properties.

Time series analyses allow detecting the dynamics of variables representing the coordinated behaviour of the system (Haken, 1987; Balagué et al., 2014; Balagué et al., 2020; Fonseca et al., 2020). Different kinematic or physiological variables have been studied with such purpose. For instance, Vázquez et al. (2016, 2021) applied Detrended Fluctuation Analysis (DFA) to the series of elbow joint angle while performing a quasi-isometric exercise until exhaustion. In this work, the elbow angle (a kinematic variable) integrated several processes (e.g., metabolic, contractile, reflex and volitional, among others). The authors evaluated the autocorrelation of the series through the Hurst (H) exponents which informed about the adaptive properties of the performer to effort accumulation.

Time series-based analyses, such as fractal or entropy analyses of heart rate, have also been used to detect the adaptability to workloads of the cardiovascular system (Gronwald et al., 2020) or pathological states (Dutta et al., 2013; Kirchner et al., 2014). According to Kelso (1997), it is challenging to adequately set the collective variables that reduce the system dimensionality and represent the innumerable physiological degrees of freedom that control every human movement.

Applying a DFA to acceleration time series of body components during slackline tasks, Montull et al. (2020; 2021) extracted information about the motor control of slackliners, notably modulated by proprioceptive properties. Consequently, the time series of acceleration, an increasingly common monitored variable in sporting contexts (Scott et al., 2016; Simperingham et al., 2016), has the potential of being an adequate kinematic variable to capture proprioception in stability and balance tasks (Montull et al., 2021).

**FIGURE 1:**
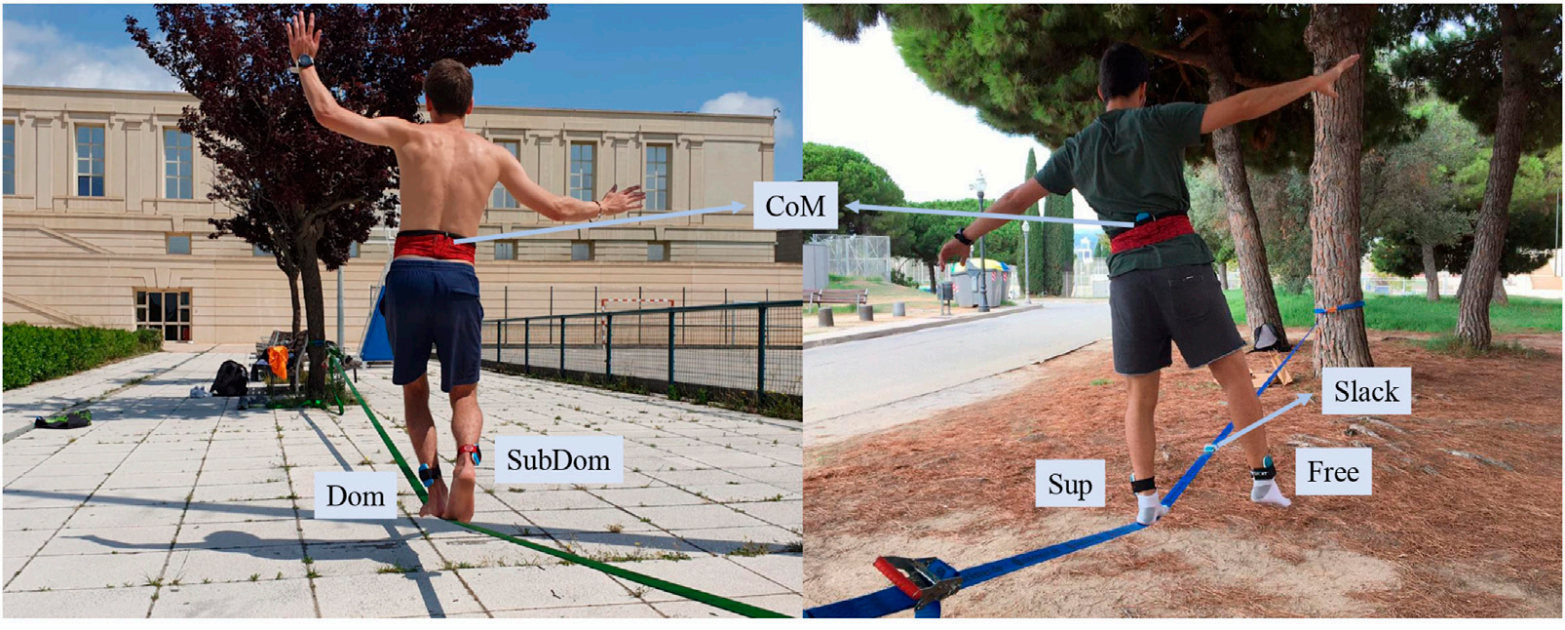
Representation of the task performed in each experiment: walking (left) and standing up balancing (right). Abbreviations of the monitored acceleration points: Dom = dominant leg; SubDom = subdominant leg; CoM = centre of mass; Sup = support leg; Free = free leg; Slack = slackline.

Slacklining is a challenging task that relies on a tight physical coupling of the performer with the environment, represented by the slackline (Montull et al., 2020). The slackline is an unstable and narrow pendulum tensioned between two anchors that requires continuous fast adjustments of body components to stand up in balance (see Figure 1) (Paoletti and Mahadevan, 2012).

H-exponents have been used to evaluate the time-variability structure of acceleration of body components of slackliners Montull et al. (2020; 2021). Ranging from 0 < H < 0.5, the fluctuations are featured by an anti-persistent character and reflect a tight and fast control related to a more adaptive behaviour (Terrier and Dériaz, 2012; Vázquez et al., 2016). In contrast, in the range of 0.5 < H < 1, the fluctuations are featured by a persistent temporal structure and relate to a more rigid motor control that may lead to the consequent impending instability (Scheffer et al., 2009; Delignières et al., 2011). These acceleration fluctuations of body components change synergistically (Haken, 1987) for stabilizing the slackline (the performance variable) (Montull et al., 2021). Thus, the slackline integrates and compresses the fluctuations of the whole performer-environment system.

This exploratory study aimed to evaluate the potential of the time-variability properties of acceleration during slackline tasks to assess proprioception and its sensitivity to training effects. We hypothesized that the H-exponent would be lower 1) in highly trained compared to poorly trained slackliners, and 2) in beginners after a slackline training process. Accordingly, we also hypothesized that slackline, the performance variable, could be more sensitive to training process than body components.

## 2 Methods

### 2.1 First experiment: Assessing proprioception of slackliners with different expertise

#### 2.1.1 Participants

Nineteen slackliners (17 males, two females, 25.3 ± 4.9 years, 70.21 ± 8.79 kg, 1.79 ± 0.05 m) of different levels (training frequency = 2.29 ± 2.98 h/week; training age = 3.05 ± 2.87 years), from a faculty of Sport Sciences and a Slackline Club, were recruited. Two training groups were formed: highly trained (*n* = 11, ≥ 2 h/week and ≥1 year) and poorly trained (*n* = 8, < 2 h/week and <1 year). As there are no specific categories to define the performance levels of slackliners, the criterion for classifying them on highly and poorly trained was based on the opinion of two expert slackliners.

To be included in the sample, the slackliners had to be able to perform the proposed slackline walking task (see procedures). The experiment was approved by the local Research Ethics Committee (072015CEICEGC) and was performed according to the ethical standards of the Helsinki Declaration. Participants were informed about the experimental procedure and signed an informed consent before the intervention.

#### 2.1.2 Procedures

Slackliners were cited individually to the mobile lab, placed in a quiet location to avoid disturbing stimuli. When arriving they were first monitored and afterwards instructed about the task as follows: “You have to walk without shoes along the slackline during 30 s at a freely chosen velocity starting close to the anchor (see Figure 1). We will notify you when the time is over. You have a maximum of 3 attempts, separated by a maximum of 5 min resting time, to accomplish the task. Any question?” The number of attempts and the resting times were fixed according to the results of a pilot study performed on a similar population.

The slackline (Gibbon Slackline TM, ID Sports, Stuttgart, Germany) was 15 m long and 5 cm wide. The tension (T) of the slackline’s anchors (5.28 ± 0.65 kN), placed at 0.85 m from the ground, was calculated through the following formula:

$$T\left(kN\right)=\left[L\left(m\right)\times M\left(kg\right)\right]/\left[S\left(m\right)\times 400\right]$$

where M is the mass of the participants, L is the length of the slackline (15 m), and S is the sag under load (ensuring at least 0.5 m in the center) (Conley, 2006; Montull et al., 2020).

#### 2.1.3 Data acquisition

Accelerometer devices WIMU PRO™ (Real Track Systems, Almería, Spain) (Gómez-Carmona et al., 2019) were placed in the legs and in the lumbar region close to the center of mass (CoM) of slackliners (see Figure 1). Through a questionnaire, slackliners reported their dominant (Dom) and subdominant (SubDom) leg. For the legs, the accelerometers were fixed on the outside part above the lateral malleolus (Mannini et al., 2013), while for the CoM on the zone of L3 (Moe-Nilssen and Helbostad, 2004; Schütte et al., 2016). The acceleration was recorded at a sampling frequency of 100 Hz. Before the measurements, the calibration of such IMUs was performed on a flat, even surface with the Z-axis perpendicular to the surface, according to the manufacturer’s specifications.

#### 2.1.4 Data analysis: DFA

DFA was applied in all acceleration time series to analyze their time-variability properties. This analysis was carried out as follows (according to Ihlen, 2012; Peng et al., 1994; 1995): Firstly, the total length of the acceleration time series (*N* = 3,072 data points) was integrated with the following equation:

$$Y\left(i\right)\equiv \overset{i}{\underset{k=1}{\sum }}\left[x_{k}-\langle x\rangle \right]$$

$x_{k:\;}$ time series of acceleration

x : average acceleration of the *N* data points

Then, the local trend was calculated to fit the acceleration time series using a quadratic polynomial function (Ihlen, 2012). The resulting time series were divided into different window scales *n* of equal length, subtracting the local trend in each window.

For all studies, root mean square (RMS) fluctuation was calculated using the following equation:

$$RMS=\sqrt{\frac{1}{N}\overset{N}{\underset{k=1}{\sum }}{\left[y\left(k\right)-y_{n}\left(k\right)\right]}^{2}}$$

$y\left(k\right)$ : integrated time series (i.e., velocity)

$y_{n}\left(k\right)$ : local trend in each box

The H-exponent, obtained as the value of the linear regression slope between the scale and the local fluctuations in a log-log diffusion plot, was used to determine the character of fluctuations for each component. Matlab^©^ R2016b was used for this analysis.

#### 2.1.5 Sensitivity of H-exponents to training expertise

The obtained H-exponents (H_Dom_, H_SubDom_, H_CoM_) were compared between highly and poorly trained groups. Mann-Whitney test and Cohen’s d were computed for this purpose. According to Cohen (1988), *d* < 0.2 means no effect, 0.2 ≤ *d* ≤ 0.5 means small effect, 0.5 ≤ *d* ≤ 0.8 means intermediate effect, and 0.8 < *d* means large effect. The level of significance was set at *p* ≤ 0.05 throughout the study. Statistical analyses were performed with SPSS v.15 (SPSS Inc., Chicago, United States).

### 2.2 Second experiment: Assessing proprioception before and after a training process

#### 2.2.1 Participants

Twelve volunteer beginners (five males, seven females, 20.6 ± 2.27 years, 65.70 ± 8.89 kg, 1.71 ± 0.08 m) with no experience at all or just introduced (less than 3 slacklining lessons or practices) were recruited. They had to be committed to following the training process from the beginning to the end (see procedures). Participants were informed about the experimental procedure and signed informed consent before the intervention.

#### 2.2.2 Procedures

As in the first experiment, all slackliners were cited individually to the mobile lab, placed in a quiet location to avoid disturbing stimuli. When arriving they were first monitored and afterwards instructed about the testing task as follows: “You have to stand up, without shoes, balancing on a slackline for at least 11 s (Figure 1). You must choose one leg as the supporting leg (Sup) and the other as the free leg (Free) (no contact with the slackline). The tiptoe of the Sup must be in the sign of 1 m from the anchor. We will notify you when the time is over. You have a maximum of 5 attempts, separated by a maximum of 5 min resting time, to accomplish the task. Any question?” The number of attempts and the resting times were fixed according to the results of a pilot study performed on a similar population. Slackline tension was calculated in the same way as in the first experiment.

The testing task was repeated before and after an individualized training process consisting of surpassing 50 challenging slackline tasks of progressive difficulty (e.g., two steps forward and standing up balancing at least 3 s, standing for 5 s together with a pair while holding the hands, walking all the slackline without falling, etc.). The testing task was not included as a challenging task. During the training process participants were requested to keep their training habits and avoid other type of proprioceptive training (stability or balance exercises). Participants could practice every challenge on their own and as many times they needed until succeeding. The success was supervised once a week. The whole training process lasted from one to 3 months (depending on the individual learning abilities) with a median (IQR) training time of 90 h (47.5 h). After surpassing the 50 challenges, they performed the post-training testing within the next 7 days.

#### 2.2.3 Data acquisition and analysis

Acceleration was captured in the same way as in the first experiment for both legs (Sup and Free) and CoM (Figure 1). The acceleration of the slackline (Slack), the performance variable (Montull et al., 2021), fixed at 0.5 m from the toe of Sup, was also registered and included in the analysis. The acceleration was recorded at a sampling frequency of 100 Hz to ensure enough data points for the DFA. The total length of the acceleration time series ranged from *N* = 1,024 to 2,048 data points. DFA was calculated as explained in the first experiment, also obtaining H-exponents as an outcome.

#### 2.2.4 Sensitivity of H-exponents to training process

The sensitivity of H-exponents to training was tested by comparing the H values of each component pre- and post-training. Wilcoxon test and Cohen’s d were used for this purpose. Previously, Kolgomorov-Smirnov test was applied to demonstrate a non-normal distribution of all variables.

## 3 Results

### 3.1 Time-variability of acceleration of body components and slackline

As illustrated in Table 1, depending on body components, the fluctuations profile changed. Differently than others, the H_CoM_ revealed, on average, a weakly anti-persistent structure of fluctuations in both experiments (H close to 0.5). In contrast, both legs and slackline had persistent structures: H_Dom_, H_Sup_ and H_Slack_ with more moderate persistent dynamics, while H_SubDom_ and H_Free_ legs tended to have higher persistent dynamics (H close to 1).

**TABLE 1:** Mean ± SD of the Hurst exponents of body and slackline acceleration from the two experiments.

		HDoM	HSubDom	HCoM	
1st experiment	Highly trained	0.63 ± 0.11	0.68 ± 0.11	0.47 ± 0.03	
	Poorly trained	0.74 ± 0.08	0.76 ± 0.11	0.50 ± 0.06	

		HSup	HFree	HCoM	HSlack
2nd experiment	Pre-training	0.67 ± 0.13	0.76 ± 0.14	0.46 ± 0.07	0.75 ± 0.15
	Post-training	0.62 ± 0.10	0.68 ± 0.14	0.47 ± 0.10	0.62 ± 0.17

Notes: H = hurst exponent; Dom = dominant leg; SubDom = subdominant leg; CoM = centre of mass; Sup = support leg; Free = free leg; Slack = slackline.

### 3.2 Effects of training on time-variability properties of acceleration

In the first experiment, the highly trained group showed lower persistent dynamics of acceleration in all body components compared with the poorly trained group (see Table 1). In particular, displaying considerably lower values of H_Dom_ (Z = −2.15; *p* = 0.03; *d* = −1.12), and intermediately lower values of H_SubDom_ (*d* = −.63) and H_CoM_ (*d* = −.57).

In the second experiment, post-training values reduced the persistency of fluctuations compared with pre-training (see Table 1). H_Slack_ was significantly lower (Z = −2.35, *p* = .02; *d* = −.81), tending towards a moderate anti-persistent profile. Also, both legs tended towards lower persistency: an intermediate reduction was found in H_Free_ (*d* = −.6) while small effect size was shown in H_Sup_ (*d* = −.43).

## 4 Discussion

This study found that H-exponents, evaluating the time variability of acceleration during slackline tasks, captured body and slackline fluctuations and were sensitive to training effects. The motor control of the different body components, favored by proprioceptive properties, showed that: CoM featured rapid and frequent fluctuations (weakly anti-persistency) for maintaining the postural control, Sup/Dom featured persistent moderate fluctuations for rapidly adjusting to slackline fluctuations, and Free/SubDom featured high persistent fluctuations for compensating through large and rigid movements the impending instability (Montull et al., 2020; 2021). All components dynamically interacted and spontaneously reorganized to accomplish the task goal (i.e., stabilize the slackline).

The time-variability properties of acceleration showed sensitivity to training effects. In the first experiment, the highly trained group tended towards lower persistent profiles (H closer to 0.5) of all body components and showed better control of both legs during slackline walking. The dominant leg showed the highest differences, probably due to its essential role in keeping the stability while walking. Such improvement in the motor control has been related to longer stability performance (Montull et al., 2021). These findings agree with previous work studying other sports activities, such as running, in which trained runners, compared to non-runners, showed lower persistency of gait cycle fluctuations (Nakayama et al., 2010).

In the second experiment, legs and slackline tended towards lower persistent dynamics after training. The slackline, representing the performance variable and compressing the proprioception of the whole performer-slackline system, reflected the largest improvement. In contrast, the time-variability of CoM’s acceleration did not show significant changes. Compared to legs, this lower sensitivity to training of CoM is probably due to its longer distance from the slackline (Singh et al., 2020).

Considering the stabilizing synergies between the body components, training should probably induce improvements at different levels and timescales, including microscopic (e.g., cell mechanics), mesoscopic (e.g., reflex processes) and macroscopic processes (e.g., attention, concentration) in a correlated way. This enhancement of motor and proprioceptive control is linked to the self-development of balance-retention and fatigue-reduction (Gabel et al., 2016).

It is plausible to suggest how the multiple embedded physiological components of slackliners interact to permit motor-proprioceptive control (Singh et al., 2020; Gabel et al., 2021; Seidel-Marzi et al., 2021). Accordingly, acceleration seems an adequate variable to describe and compress performers’ behaviour in stability and balance exercises. This type of kinematic variables, capturing the action level, is promoted by the Network Physiology of Exercise because they provide integrated information about the synchronization and coordination of processes interacting horizontally (e.g., within the same level) and vertically (among levels, e.g., cells, tissues, organs, etc.) in the organism (Balagué et al., 2020; 2022). Therefore, the proposed proprioceptive assessment might be particularly helpful to avoid fragmented, oversimplified, passive and decontextualized proprioception measures capturing this property in a reasonably integrative and applied manner (Ager et al., 2017; Muñoz-Jiménez et al., 2021). In addition, it may provide sensitive information about the rapid, sub-conscious, and often imperceptible motor readjustments that, modulated by proprioception, operate in real exercise contexts (Hillier et al., 2015).

Diverse balance and coordinative activities, dependent on proprioceptive abilities, might also be assessed objectively by analysing the variability properties of acceleration. This opens an exciting research line to approach different phenomena related to health and performance. For example, to assess the state of neurodegenerative disorders (Lheureux et al., 2020), the acute effects of fatigue (Montull et al., 2019), the risks of sports injuries (Pol et al., 2019) or the rehabilitation progress (Lheureux et al., 2020).

This is an exploratory study and further research is needed to robustly claim that proprioception can be assessed through time-variability properties of acceleration. In particular, exploring the validity and reliability of acceleration time series and DFA in other types of exercise is warranted. Synergies analysis, such as Uncontrolled Manifold, should also be considered as a complement to understand better the proprioceptive communication between the studied components in balance exercises (Montull et al., 2021).

## 5 Conclusion

This study suggests that the time-variability structure of acceleration in real contexts, like slackline tasks, may objectively measure proprioception and be sensitive to its training effects. From a practical point of view, the monitorization of the slackline itself, compressing the motor synergies in this type of task, seems to be sufficient to inform about the proprioceptive control.

## Data Availability

The raw data supporting the conclusion of this article will be made available by the authors, without undue reservation.

## Abbreviations

CoM, Center of mass; DFA, Detrended Fluctuation Analysis; Dom, Dominant leg; Free, Free leg; H-exponent, Hurst exponent; Slack, Slackline; SubDom, Subdominant leg; Sup, Support leg.
